# Role of Nephropathological Factors with Fibrinogen in the Progression of Human Diabetic Nephropathy in the Pakistani Population

**DOI:** 10.2174/0115733998433035251219191845

**Published:** 2026-02-13

**Authors:** Maha Maqsood, Saima Sharif, Muhammad Saqib, Muhammad Maqsood, Shagufta Naz, Saira Rafaqat, Ali Hasan

**Affiliations:** 1 Lahore College for Woman University, Department of Zoology, Lahore, Pakistan;; 2 Department of Medicine, Sir Ganga Ram Hospital, Pakistan;; 3 DHQ Teaching Hospital Gujranwala, Civil Lines, Gujranwala, Pakistan;; 4 Department of Zoology, Wildlife and Fisheries University of Agriculture Faisalabad Constituent college, Toba Tek Singh, Pakistan

**Keywords:** Diabetes mellitus, diabetic nephropathy, fibrinogen, pakistani population, GFR, gene expression

## Abstract

**Introduction:**

Fibrinogen is synthesized in the liver and is a marker of acute-phase inflammation. It is essential for regulating the inflammatory response by attaching to receptors on the surfaces of many cells and is involved in the pathogenesis of kidney diseases. The objective of this investigation is to identify the role of fibrinogen in the progression of diabetic nephropathy in the Pakistani population.

**Method:**

This study was based on an analytical comparative research. A total number of 1152 subjects were enrolled from selected cities of Pakistan (Islamabad, Lahore, Multan, Gujranwala, Karachi, and Gharro). The subjects were classified into the Control group (n=384), the Diabetes Mellitus group (DM) (n=384), and the Diabetic Nephropathy group (DN) (n=384) based on glycemic condition and glomerular filtration rate. The serum fibrinogen level was assessed using an enzyme-linked immunosorbent instaed of test its assay (ELISA). The expression profile of fibrinogen was determined using real-time PCR.

**Result:**

The highly significant difference in serum fibrinogen levels was found among the control, DM, and DN groups. The fibrinogen expression was increased by more than ~ 12.03 n-fold in the control group as compared to the DM group (n-fold = ~ 4.78) and the DN group (n-fold = ~ 5.42). When the correlation between the serum fibrinogen and blood sugar was analyzed, the DN (r=-0.145) had an inverse and highly significant relationship. The fibrinogen expression has a direct and significant correlation with blood urea (r=0.166). The stepwise linear regression analysis concluded that expression of fibrinogen is an important determinant of kidney function in the DM group. Creatinine, age, and blood sugar were important determinants in the DN group.

**Discussion:**

The cities of Pakistan, *i.e*, Lahore, Gujranwala, Multan, Islamabad, Karachi, and Gharro, exhibited significant variations in expression of Fibrinogen. This highlights a notable difference in the genetic profile across these regions of Pakistan.

**Conclusion:**

The results of our study indicate a complex relationship between levels of fibrinogen and diabetic nephropathy. The findings suggest that in diabetic renal disease, fibrinogen could have an early role, whereas with the progression of nephropathy, its significance diminishes. Further studies are required to explain the underlying mechanisms involved, relating this as a potential target for therapy in diabetic kidney disease.

## 1 INTRODUCTION

Type II diabetes mellitus (T2DM) is characterized by impaired glucose tolerance, through which insulin resistance is associated. Insulin insufficiency may impact the body's muscle, liver, and adipose tissues in terms of glucose utilization. The progression of insulin resistance and type II diabetes is significantly influenced by reduced glucose tolerance as well as other factors such as genetic predisposition, environmental factors, nutrition, lack of physical activity, and obesity [1].

Individuals with diabetes (type 1 and type 2) may develop diabetic nephropathy (DN) when their kidneys undergo certain pathologic structural and functional alterations as a result of their kidneys' interactions with diabetes mellitus (DM). Proteinuria, hypertension, and persistent declines in kidney function are among the clinical symptoms manifested by these modifications [2].

Fibrinogen is an essential component in the inflammatory response and blood coagulation. When combined with thrombin, fibrin, the primary component of blood, forms a clot [3]. Fibrinogen is present in the blood at quantities ranging from 2 to 4 g/L under normal circumstances, and its half-life is about 4 days. The fibrinogen concentration in the blood, however, increases in pathological conditions such as after injury or in diseases linked to vascular disruption, infection, or inflammation. Therefore, fibrinogen is thought of as a reactant in the acute phase [4].

Fibrinogen plays a critical role in determining the severity of local or systemic inflammation, as evidenced by research in genetics and pharmacology. The role of fibrinogen is changing from that of a marker of vascular rupture to that of a multifaceted signaling molecule with a broad range of functions, as cellular and molecular mechanisms for fibrinogen functions in tissues are discovered. These processes have the power to alter the balance that exists between thrombosis, coagulation, fibrosis, hemostasis, defense against infection, widespread inflammation, and ultimately life and death [5].

Few studies have examined the connection between serum fibrinogen and DN, even though a number of clinical and nephropathologic factors, such as proteinuria, e-GFR, and glomerular and tubulointerstitial lesions, have been associated with poor kidney health [6]. In cross-sectional studies, serum fibrinogen levels were associated with decreased renal function, proteinuria, and glomerular basement membrane (GBM) thickness [7].

Examinations of the function of inflammatory mechanisms in diabetes and its complexities depend on the initiation and advancement of infection. Such an improved comprehension of the fundamental cause of diabetes could be useful in introducing novel treatment strategies alongside currently used non-pharmacologic and pharmacologic interventions. The objective of this research is to point out the role of fibrinogen in the development of diabetic nephropathy.

## 2 MATERIALS & METHODS

This case-control study was conducted in different hospitals of Punjab (Lahore, Gujranwala, Multan, Islamabad) and Sindh (Karachi and Ghaaro) from January 2018 to December 2021. This study aimed to determine the role of fibrinogen in diabetes and diabetic nephropathy in the Pakistani population. Age, genetics, food, sedentary behavior, environment, and physical exercise were some of the perinatal factors that are being studied in these cities.

### 2.1 Study Population

The study subjects were systematically recruited from patients attending the Diabetes and Nephrology Departments of Punjab and Sindh, including different hospitals. The Punjab province covered the following hospitals: Sheikh Zayed Hospital, Lahore; DHQ Hospital, Gujranwala; Nishtar Hospital, Multan; and the Federal General Hospital, Islamabad (Diabetes OPD and Nephrology Ward). Patients from the Sindh province were recruited from Civil Hospital, Karachi, and Shaikh Zaid Medical Center, Mirpur Sakro, Ghaaro (Diabetes OPD and Nephrology Ward). Participants from these places were enrolled after they had provided their informed consent to participate in the study.

### 2.2 Sample Size

The sample size was calculated by the Rao software formula, which was based on the prevalence of diabetes in Pakistan (26.3%) [8]. A total of 1152 individuals were registered in the study. Based on selection criteria, the study subjects included 384 healthy individuals (Control Group) with no family history of diabetes and nephropathy, 384 subjects with type 2 diabetes (Fasting blood glucose > 126mg/dl) without nephropathy (DM Group), and 384 diabetic nephropathy patients (DN Group). To ensure representation from diverse geographical locations, samples were collected from six different cities (Lahore, Gujranwala, Multan, Islamabad, Karachi, and Ghaaro). This approach allowed us to maintain homogeneity within each group while capturing regional variations.

### 2.3 Selection Criteria

The nephrologist's renal examination served as the basis for the nephropathy criterion. Participation was predicated upon people having first, second, or third stage nephropathy and having type 2 diabetes for at least ten years. Conditions such as being above the age of 80, having a history of malignancy, having a hematological disease, or having a cardiovascular problem were not considered for inclusion in this study. The participants who had a history of malignancy, hematological diseases, or cardiovascular illness were not included.

### 2.4 Study Design

This study was based on an analytical comparative research. In this research, we compare the serum and fibrinogen expression of healthy people with DM and DN subjects. Purposive sampling with non-probability was the basis for this research.

### 2.5 Collection of a Blood Sample

Blood samples were collected from subjects suffering from DM and DN at their respective hospitals. Blood samples were collected into ethylenediaminetetraacetic acid (EDTA)-coated tubes and serum separation tubes. EDTA-coated blood samples were used for the extraction of RNA within 2-4 hours of collection. To extract the serum, the samples were centrifuged for 15 minutes at 3000 rpm after 30 minutes. The serum and RNA samples were kept at -80°C until analysis.

### 2.6 Biochemical Assessments of Renal Function Parameters

A chemical analyzer (ERBA Chemistry Analyzer, Model # CHEM-7, Serial # 9047) was used to perform the measurements of serum creatinine (Cat No.CS.604), blood urea (Cat. No. CS612), and uric acid (Global Uric Acid Kit/PAP method).

### 2.7 Assessment of Blood Urea Nitrogen (BUN)

Blood Urea Nitrogen (BUN) measures the urea nitrogen component. BUN is used to assess the renal and liver function of the body. Using a conventional formula, the blood urea nitrogen (BUN) was assessed.

To calculate BUN from total blood urea concentration = 


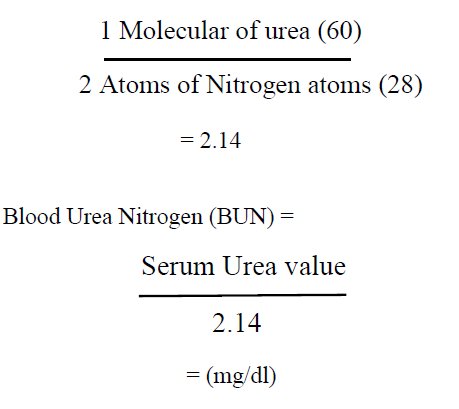


### 2.8 Assessment of Glomerular Filtration Rate (GFR)

Using a standard procedure, the creatinine-based glomerular filtration rate (GFR) was calculated.

Female: GFR = 144 × (Scr/61.9)^-0.329^ × (0.993)^Age^

Male: GFR = 141 × (Scr/79.6)^-0.411^ × (0.993)^Age^

### 2.9 Assessment of Lipids Profile Parameters

The lipid profiles (Triglycerides, total cholesterol, High Density Lipoprotein Cholesterol, Low Density Lipoprotein Cholesterol) measurements were performed with the help of a chemistry analyzer by using commercially available kits.

### 2.10 ELISA Assay for Fibrinogen

A Human Fine Test Enzyme-Linked Immunosorbent Assay Kit (catalog number EH3057) that is available for acquisition from Wuhan Fine Biotech Co., Ltd. was utilized to determine the amounts of fibrinogen in the serum. To determine, (the relative O.D.450) = (the O.D.450 of each well) - (the O.D.450 of the blank well). By graphing the concentration of each standard solution (Y) against its relative O.D.450, the standard curve (X) is derived. To determine the sample's target concentration, extrapolate the standard curve. The Curve Expert 1.3 / 1.4 program was used to perform the calculations.

### 2.11 RNA Extraction & Quantification

To analyze gene expression, 5 ml of peripheral blood was obtained from control, DM, and DN patients in EDTA- coated tubes, and RNA was extracted by using the Trizol procedure (Rio, 2010). Nano drop (Multiskan SkyHigh Microplate Spectrophotometer) was used to measure both the quantity and quality of RNA.

### 2.12 Primer Designing

The human fibrinogen sequence was obtained from the National Center for Biotechnology (NCBI) and examined using the bioinformatics tools (www.genome.ucsc.edu) IN-SLICO and BLAST (basic local alignment program). To prevent genomic DNA impurities, primers were designed using exonic sequences. For primer design, the sequences were entered into the primer scheming application (www.primer3) (Table **1**). The primer was prepared by a readily available commercial industry. Primer optimization was performed to ensure the working temperature of primers. Glyceraldehyde 3-phosphate dehydrogenase (GAPDH) was used as a reference (housekeeping gene).

### 2.13 cDNA Synthesis & Gel Electrophoresis

The first strand cDNA was synthesized from RNA using the Maxima® First Strand cDNA Synthesis Kit (Thermo Scientific) (#K1622). To assess the quality of the PCR product, the Gel Electrophoresis technique was performed.

### 2.14 Real-Time PCR for Expression Analysis

The relative gene expression study of fibrinogen was also performed utilizing the Applied Biosystem with Step One Software V2 of RT-PCR. Maxima SYBER Green Master Mix (CAT # K0221) was used according to the manufacturer's instructions. The expression of the target gene was normalized by utilizing the GAPDH gene as a reference value. RT PCR cycling conditions include one cycle of 94°C for 4 minutes, followed by 30 cycles of 94°C for 30 sec, Tm (gene) for 20-30 sec, and 72°C for 45 sec. The final extension was at 72°C for 5 min.

### 2.15 Statistical Analysis

The demographics data were reported in the form of mean ± SD. The data were checked for normal distribution, and parameters that were not normally distributed were transformed for normality before performing statistical analysis. Analysis of variance (ANOVA) tests were conducted to compare the mean values of the control, diabetes, and diabetic nephropathy groups. The Pearson correlation coefficient was employed to determine the association between the studied variables. The impact of variables on the progression of DN was investigated using stepwise multiple regressions. Applied Bio-System estimated the CT value of genes using Step One Software V2 of RT-PCR. Data Assist Software was used to examine the CT value of RT-PCR. Gene expression data was expressed as fold change or relative levels using the comparative CT value (2^−ΔΔCT^). Statistical package for social sciences (SPSS) software was used for the determination of the level of significance. The degree of significance was determined at the 95% and 99% probability levels.

## 3 RESULTS

### 3.1 Demographical Characteristics

This study was done to assess the role of fibrinogen in diabetic nephropathy of the Pakistani population. A questionnaire was used to collect demographic information such as family history of DM, family history of diabetic nephropathy, smoking status, cardiac surgery, hypertension and congenital heart diseases, and duration of diabetes since they were suffering. (Table **2**). Significant differences was observed among the studied groups regarding age, glycated haemoglobin (HbA1c), systolic blood pressure (SBP), Diastolic blood pressure (DBP), blood urea, blood sugar, serum creatinine, BUN, GFR, uric acid, Body mass Index (BMI), Wait to Hip Ratio (WHR), HDL, TG and LDL (Table **3**). Significant differences were not observed between the control, diabetic (DM), and diabetic nephropathy (DN) groups in relation to TC (Table **3**).

### 3.2 Assessment of Fibrinogen in the Studied Subjects

The serum levels of fibrinogen considerably differed among the studied groups (16.22 ± 28.43 ng/ml, 4.79 ± 9.52 ng/ml, 11.93 ± 37.41 ng/ml) (Table **3**). The relative expression of the fibrinogen gene was quantitatively represented as an n-fold difference compared to the reference gene (GAPDH). The fibrinogen expression profile was increased by Σ12.03 fold in the Control group and by Σ5.42 fold in the DN group, as compared to Σ4.75 fold in the DM group, indicating a highly significant difference (Fig. **1**, Tables **S1**, and **3**).

### 3.3 Correlation Analysis of Serum and Fibrinogen

The association between serum fibrinogen and indicators of renal impairment was assessed using Pearson's correlation method. When the association between blood fibrinogen and blood sugar was investigated, the DM (r=-0.078) group had an inverse relationship when compared to the control and DN groups. In the DM group, a direct and highly significant association was seen between serum fibrinogen and BUN (r= 0.135**) in comparison to the other groups. Similarly, a direct and very significant correlation was found with blood urea in the DM group (r= 0.135**) as compared to the control (r= -0.016) and DN (r= 0.003) groups (Table **4**).

### 3.4 Correlation Analysis of Expression of Fibrinogen (Fold Change)

In the correlation analysis of fibrinogen expression, a direct and highly significant link was found with blood urea (r= 0.166**) in the diabetic nephropathy group when compared to the control and diabetes mellitus groups. The inverse correlation between fibrinogen expression and GFR was detected in the DN (r=-0.068) and control (r=-0.016) groups; however, the direct association was observed in the DM (r=0.039) group. In the DM group relative to the DN group, an inverse correlation was observed between fibrinogen expression and blood sugar (r=-0.002), blood urea (r=-0.057), and creatinine (r=-0.064) (Table **4**).

### 3.5 Stepwise Multiple Regression Analysis

Stepwise multiple regressions were employed to ascertain the association between GFR, the dependent variable, and serum fibrinogen, along with fibrinogen expression as independent variables. The severity of nephropathy had been evaluated by kidney function tests, with the glomerular filtration rate (GFR) being the most reliable. This is why we selected GFR as the dependent variable in our analysis.

In the control group, four (3) models were generated. These models revealed that age, creatinine levels, and uric acid levels were significant predictors of GFR in the control group (Table **5a**). When stepwise multiple regression was used in the DM group, eight (8) models were generated, suggesting that creatinine levels, age, BMI, serum fibrinogen, DBP, SBP, uric acid, and fibrinogen expression were significant indicators of kidney function (Table **5b**). In the DN group, three models were computed, indicating that creatinine, age, and blood sugar were significant factors determining the etiology of diabetic nephropathy (Table **5c**).

### 3.6 District-wise Analysis of Studied Population (Lahore, Karachi, Gujranwala, Multan, Islamabad, & Gharro)

When the data was evaluated based on geographical distribution in selected Pakistani cities (Lahore, Gujranwala, Karachi, Multan, Islamabad & Gharro). The populations under study areas showed highly significant differences in BMI, WHR, HbA1c, SBP, DBP, blood sugar, HDL-C, TG, LDL, blood urea, creatinine, BUN, GFR, and uric acid (Table **6**). It was observed that patients with diabetic nephropathy in Karachi tend to be older. As a consequence, a significant age difference was observed in the Karachi population when compared to other selected cities. The significant differences of serum fibrinogen in the Multan population were observed in the DN group as compared to other selected cities (Table **6**).

When the data of cities were assessed, a significant direct correlation between fibrinogen and BUN was identified in the Gujranwala (r=0.319*) population and the Multan (r=0.257*) population, as compared to other cities. The highly significant association was observed in the Karachi population (r=0.406**) regarding blood sugar and fibrinogen as compared to Lahore, Gujranwala, Multan, Islamabad, and Gharro populations (Table **7**).

When the expression profile of fibrinogen was compared in the DN group, the fibrinogen expression was increased ~ 16.43 fold in the Islamabad population as compared to Lahore (*n*-fold= ~ 0.78), Gujranwala (*n*-fold= ~ 10.21), Karachi (*n*-fold= ~ 5.30), Multan (*n*-fold= ~ 3.79), and Gharro (*n*-fold= ~ 0.74) populations (Fig. **2**).

## 4 DISCUSSION

Fibrinogen is a macromolecular glycoprotein that is made by the liver and has a high blood concentration compared to any other coagulation factor. It has a dynamic balancing function in fibrinolysis and blood coagulation, promoting normal blood circulation and controlling platelet aggregation and the activity of vascular endothelial cells [9]. This study aimed to evaluate the relationship of fibrinogen levels with nephropathological factors in diabetes and diabetic nephropathy within the Pakistani population.

Fibrinogen appears to be a unique pathogenic activator of fibroblasts involved in renal fibrosis. Fibrinogen promotes renal tubulointerstitial fibrosis. Fibrinogen primarily acts to occlude blood arteries and thereby prevent excessive bleeding [10]. Diabetic nephropathy progression is expedited by an assortment of modifiable and non-modifiable risk factors, related comorbidities, and unhealthy lifestyles such as physical inactivity, unregulated blood pressure, and blood glucose [11].

DN often follows a latency phase that may occur for many years in approximately one-third of individuals with diabetes. The necessity of examining individuals for microalbuminuria versus screening for diabetic nephropathy remains a topic of debate. This modified medical approach aims to allocate resources for more extensive therapy and early preventive measures specifically to those at the highest risk [12].

Diabetic nephropathy is a complication that develops over time, typically after prolonged exposure to hyperglycemia. In our study, patients with diabetic nephropathy are older and have a longer duration of diabetes compared to those without nephropathy. Despite age adjustment, there was a significant difference between the ages of the diabetic subjects and those with diabetic nephropathy; the latter were about 2-3 years older. This implies that other factors aside from age are responsible for the development of diabetic nephropathy. Baek *et al*., (2021) indicated that patients with type 2 diabetes mellitus (T2DM) and those with type 1 diabetes mellitus (T1DM) encounter complex interactions among age, age of diagnosis of disease, and duration of diabetes in assessing their risk of diabetic complications [13]. Based on earlier national and international research, age plays a significant role in the increased risk of pre-diabetes and diabetes. Age had a significant impact on the risk of diabetes, especially after accounting for other confounders [14]. The population-attributable risk percentage (PAR%) showed a decline with age, decreasing from 56.6% in participants aged 40 to 55 to 47.4% in those aged 75 and above with pre-diabetes, where it reached 65.5% [15].

In this study, a highly significant difference in HbA1c was detected in the DM group relative to the DN group may be because the diabetic nephropathy group takes medication for maintaining their glucose levels, since hyperglycemia promotes renal failure, thus responsible for the variance. Demirelli *et al*., claim that the correlation between changes in HbA1c and the onset of chronic kidney disease in individuals with type 2 diabetes suggests that both the average stability and variability of glycemic control affect renal health. Based on the accessible data, decreasing fluctuations in HbA1c may provide additional protection against the progression of micro-vascular complications, including those that affect renal function [16].

Findings of this study indicated that the BMI of DN was significantly lower than that of DM, as reported by Hu and Zang (2020) that among patients with type 2 diabetes who are overweight, HbA1c serves as a risk factor for diabetic nephropathy (DN). The results imply that the patient's weight loss may have been caused by the initial manifestation of nephropathy. Among Chinese patients with biopsy- confirmed diabetic nephropathy, 22.3% were classified as obese, while 53.7% were overweight/obese. The incidence of obesity in individuals with early-stage DN was as high as 24.8% higher than in the overall Chinese population [17].

Hypertension, commonly observed in patients with diabetes and renal disorders. Mohammad *et al*., indicated that hypertension increases the risk of cardiovascular morbidity and mortality along with the onset and advancement of kidney disease. Numerous studies have demonstrated the possible relevance of blood pressure reduction in the treatment of nephropathy [18]. In this study, a highly significant difference in blood pressure was observed in the DN group when compared to the DM and control groups. The majority of subjects in the DN group either have high blood pressure or are undergoing anti-hypertensive therapy. A study conducted by Van Buren indicated that individuals with type 2 diabetic nephropathy demonstrate increased levels of hypertension, and it is recommended that the goal of blood pressure management should be to attain a blood pressure of less than 130/80 mm Hg [19].

This study demonstrated that participants with diabetic nephropathy exhibited elevated levels of blood urea, serum creatinine, and blood urea nitrogen in comparison to those with diabetes mellitus and the control group. The study indicated a reduction in glomerular filtration rate (GFR) in diabetic nephropathy (DN) subjects with uncontrolled serum creatinine levels when compared to both control and diabetes mellitus (DM) subjects. This may cause deterioration of renal function. In diabetic subjects, the blood's serum creatinine level can be an efficient prognostic marker and predictor of renal impairment. A cross-sectional study conducted throughout Thailand revealed that nearly 40% of T2DM patients exhibited diminished GFR. The risk of a reduced GFR was independently associated with advanced age, albuminuria, hyperuricemia, hypertension, HbA1C 6% and HbA1C >7% [20].

GFR is influenced by multiple processes, which include those related to myogenic, tubuloglomerular feedback, neuronal, hormonal, sympathetic neurons, and angiotensin II. A reduction in GFR, which causes kidney failure, and the start of nephropathy, which can be triggered by similarities in any one of these pathways [21]. Angiotensin receptor blockers: GFR gets reduced by NSAIDs and ACE inhibitors. Prostaglandin synthesis is inhibited by NSAIDs. Since angiotensin II typically causes the efferent arteriole to constrict, ACE inhibitors, sometimes referred to as angiotensin receptor blockers, induce the afferent arteriole to widen. Numerous exogenous forms of medication (ACEI, ARBs, and NSAIDS) reduce GFR and cause renal injury [22].

The current study found that uric acid levels were elevated in patients with diabetes mellitus compared to those with diabetic nephropathy. The liver produces uric acid, which the kidney's glomerulus may readily filter when it is soluble (monosodium urate). Fractional excretion of uric acid is only 10% due to reabsorption of uric acid by renal proximal tubular cells. Hyperuricemia occurs when your blood uric acid level exceeds 6 mg/dL. Hypouricemia can be brought on by either an excessive amount of production or an inadequate amount of secretion, or both. A condition known as diabetic nephropathy (DN) can be caused by persistently elevated amounts of uric acid, which also leads to inflammation [23]. Elevated blood uric acid levels in type 2 diabetics may contribute to diabetic nephropathy. The risk of renal tubulointerstitial injury was reduced in DN patients whose glycemic control had been achieved with medication when their uric acid levels were reduced. Numerous consequences related to uric acid have been linked, some of which may contribute to the development of DN. These comprise increased RAAS activity, endothelial dysfunction, development of the inflammatory cascade, and pro-fibrotic cytokine activation [24]. Angiotensin-converting enzyme inhibitors (ACEIs) and angiotensin receptor blockers (ARBs) are two of the most significant RAAS inhibitors that have been associated with a reduction in uric acid levels and an acceleration of nephropathy pathogenesis [25].

In this study, a non-significant difference was found in total cholesterol level among the studied groups. The DN group showed an elevated level of TC in comparison to the DM and control groups. It is widely recognised that hypercholesterolaemia affects podocyte function and structure, hence aggravating renal failure. One of the reported pathways leads to glomerular injury by inducing the formation of foam cells, which are connected with eventual glomerulosclerosis and interstitial injury. Hypercholesterolaemia levels have been related to kidney fibrosis, inflammation, and triggering the innate immune response [26]. A significant difference was observed in HDL-C, LDL-C, and TG levels among the groups, with increases in HDL-C, LDL-C, and TG levels in the DN group compared to the DM and control groups, attributed to disease severity.

Fibrinogen is a 340-kDa soluble protein with serum levels of about 2.0-4.0 g/L that is mostly produced by the liver and is essential for hemostasis. Furthermore, fibrinogen has been linked to inflammation and has a significant pathophysiologic effect in accelerating the progression of renal disease. Serum fibrinogen is an acute-phase inflammatory biomarker that is becoming recognized for its significant role in modulating the inflammatory response [27].

In this research, a highly significant difference in serum and fibrinogen expression across the study groups was observed. The increased regulation of serum fibrinogen was found in the control group in comparison to DN and DM. On the other hand, fibrinogen was also overexpressed in the control group compared to the DM and DN groups, which is contrary to our study, possibly because fibrinogen levels may be influenced by the medications used to control blood glucose levels in the diabetic group, which can impact fibrinogen production. As diabetic nephropathy progresses, inflammation becomes more pronounced, and the medications may not be sufficient to suppress this inflammatory response. This could contribute to the increased fibrinogen levels observed in our diabetic nephropathy group compared to the diabetes group. Vascular injury from renal failure may be the cause of the non-significant difference in serum fibrinogen levels between individuals with DM (4.79 ng/ml) and those with DN (11.93 ng/ml). This triggers the elevated levels of fibrinogen to be produced, hence initiating the signaling pathways that contribute to the fibrin clot attributes.

Vilar *et al*., (2020) claim that various pathways contribute to the fibrinogen-disease attachment.

These include altering the usual range of fibrinogen levels or its structure, as well as initiating signalling pathways within specific physiological conditions. The latter may be engaged in numerous pathogenic pathways by altering the characteristics of fibrin clots, which can impede the binding of thrombin and plasminogen [28]. As a result, DN individuals in the Pakistani community had higher levels of fibrinogen than DM subjects.

According to our findings, a statistically significant relationship between blood urea and expression of fibrinogen was observed in the DN group. According to Yu *et al*., (2020), in cross-sectional investigations, the blood fibrinogen levels were linked with decreased proteinuria, renal function, and glomerular basement membrane (GBM) thickness [29]. Poor renal outcomes have been associated with numerous clinical and nephropathological factors, including proteinuria, e-GFR, and glomerular and tubulointerstitial lesions. Nevertheless, there are relatively few reports on the relationship between DN and serum fibrinogen, which is one of the hemostatic markers [30].

Using linear regression analysis, we evaluated the kidney parameters linked with the progression of diabetic nephropathy (DN) and concluded that blood sugar, age, and serum creatinine are important factors in the disease's progression. Likewise, there is a strong, very significant positive association between blood sugar levels and diabetic nephropathy. The predictive model developed through linear regression analysis in the DM group indicated that the likelihood of developing type 2 diabetes mellitus and insulin resistance is affected by several factors, including creatinine levels, age, body mass index (BMI), blood pressure, serum fibrinogen, uric acid, and fibrinogen expression. These are all accountable for the development of diabetic complications and hyperglycemia. In the DN group, the relationship is not so strong between fibrinogen and nephrological parameters. However, the correlations between fibrinogen and other biomarkers, such as blood urea, BUN, might be influenced by the underlying pathophysiology of the disease. A diabetic either produces inadequate insulin or their insulin does not digest glucose well. The body can metabolize glucose from food and use it as energy, owing to the hormone insulin. When an individual develops insulin resistance, the glucose accumulates in the bloodstream because it is unable to enter their cells to produce energy. Excessive blood sugar levels can harm many different tissues and organs that are necessary to maintain a normal blood pressure, which is another factor contributing to diabetic complications.

According to our study, fibrinogen plays a key role in renal fibrosis and pathological stimulator of fibroblasts. Renal fibrinogen deposition is present in many forms of chronic kidney disease, and it is believed to be a significant factor in the advancement of renal fibrosis and numerous other problems associated with diabetes [31]. Reduced glomerular basement membranes (GBMs), which cause DN, higher proteinuria, and impaired renal function, have all been linked to low blood levels of fibrinogen [32]. Furthermore, plasma fibrinogen is associated with the reduction of eGFR in subjects with CKD stages 3 and 4 [33].

Several effects of uric acid have been linked together, some of which have been connected to the onset of DN. These include RAAS activation, development of inflammatory cascades, endothelial dysfunction, and pro-fibrotic cytokine activation. Each of these impacts has been demonstrated to contribute to the progression of microvascular disease, which induces renal impairment in diabetic nephropathy [34].

After analyzing the city-wise data, the up-regulation of serum fibrinogen level was only observed in the Multan population of DN subjects as compared to Gujranwala, Lahore, Islamabad, Gharro, and Karachi. Additionally, DN participants had higher fibrinogen expression than DM and control subjects in the Multan population. We suggest these disparities may be explained in part by the variation in our research sample. The primary factor in this study that was not significant across all Pakistani cities was age. Because diabetic nephropathy progresses slowly depending on the severity of the disease, there may be a significant age gap between the DN and DM groups.

A highly significant difference was observed across all cities with Pakistani populations concerning HbA1c, blood glucose, uric acid, blood urea, systolic blood pressure (SBP), diastolic blood pressure (DBP), creatinine, blood urea nitrogen (BUN), glomerular filtration rate (GFR), high- density lipoprotein cholesterol (HDL-C), triglycerides (TG) and low-density lipoprotein cholesterol (LDL-C) except age and total cholesterol. In city-wise correlation analysis, Fibrinogen and renal variables exhibited varying associations with parameters depending on the Pakistani population's city. The main cause of the variations in the demographic and clinical data of the populations of various cities is the lifestyle, food habits, genetics, sedentary behavior, and climate differences among their populations.

A significant priority for nephrologists is to protect diabetic patients against the onset of renal failure. The advancement of future treatments for diabetic nephropathy requires the creation of innovative therapeutic strategies that focus on the core pathways responsible for renal damage, given the intricate nature of this condition.

## 5 STUDY LIMITATIONS

This research possesses particular limitations. The cross- sectional design limits the capacity to determine causal correlations between fibrinogen levels and the advancement of diabetic nephropathy. Second, while samples were taken from multiple cities in Pakistan, the findings may not be applicable to the overall population due to possible selection bias as well as limited sample diversity. Finally, molecular analyses were restricted to expression profiling, requiring functional studies to clarify the specific mechanistic role of fibrinogen in disease progression.

## CONCLUSION

This research highlights a significant association between fibrinogen levels and the advancement of diabetic nephropathy within the Pakistani population. The variations observed in various cities may be potentially linked with genetic and ethnic backgrounds. Fibrinogen predicts diabetic kidney disease in the diabetic group rather than the diabetic nephropathy group; the contradiction suggests that the relationship between fibrinogen and renal progression is not straightforward. This may imply underlying mechanisms that will need further studies to elucidate, and opens new perspectives in considering fibrinogen as a therapeutic target in diabetic kidney disease.

## Figures and Tables

**Fig. (1):**
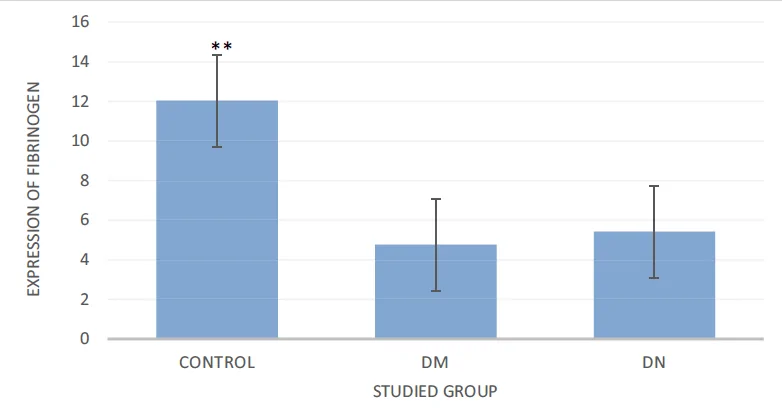
Expression of Fibrinogen in Studied Groups. (*p* < 0.01** considered highly significance difference among the groups).

**Fig. (2):**
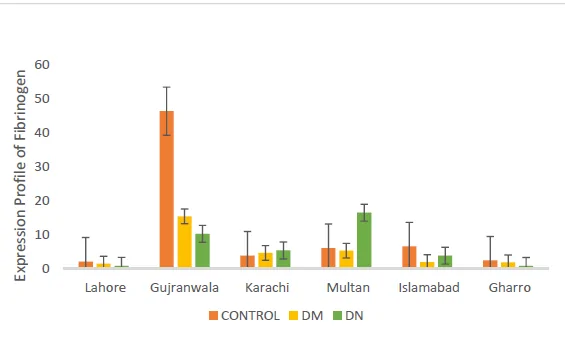
Expression profile of fibrinogen in selected cities of Pakistan.

**Table 1:** Sequence of primers used in gene expression analysis.

**Genes**	**Primer Sequences**	**Tm**	**Product Size**
Fibrinogen	F= TGCATGGAAACATGCAAAAT R= CGAAAACAAAAGCTCCCTCTT	(50 °C)	426bp
GAPDH	F: ATCCCATCACCATCTTCCAGGA R: CAAATGAGCCCCAGCCTTCT	(59 °C)	122bp

**Table 2:** Demographic data of control, diabetes mellitus and diabetic nephropathy groups.

**Parameters**	**Control Group (n=384)**	**DM Group (n=384)**	**DN Group (n=384)**
History of smokers n (%)	-	45(11)	103(27)
Family history of DM n (%)	36 (09)	320(83)	365(95)
History of DN n (%)	36(09)	320(83)	257(70)
Presence of hypertension n (%)	NIL	119(31)	243(62)
Presence of cardiac surgery n (%)	NIL	NIL	4(1)
Congenital cardiac disease n (%)	NIL	NIL	NIL
An additional types of illness n (%)	NIL	NIL	NIL
Disease duration of diabetes (years)	NIL	4-15 years	10-25 years

**Table 3:** Clinical and biochemical characteristics of control, diabetes mellitus and diabetic nephropathy groups.

**Variables**	**Control (n=384)**	**DM group (n=384)**	**DN group (n=384)**
Age (years)	48.89 ±12.76	49.23±11.1	52.67±10.22**
HbA1c (mmol/mol)	4.71 ±0.75	9.49±1.61	7.91±0.68**
S.B.P (mmHg)	110.96 ±8.50	131.12±18.72	107.45±27.9**
D.B.P (mmHg)	85.09 ±6.12	83.26±7.62	106.18±23.07**
Blood Sugar(mg/dl)	110.63±21.02	270.41±85.76	187.77±70.79**
Blood Urea (mg/dl)	23.54 ±9.25	43.90±11.64	128.80±34.90**
Serum Creatinine (mg/dl)	0.519±0.17	0.97±0.379	2.04±0.58**
BUN (mg/dl)	11.00±4.32	20.51±5.44	60.18±16.30**
GFR (mL/min/1.73m^2^)	126.11±29.03	82.76±32.05	35.76±12.72**
Uric Acid (mg/dl)	6.07±1.95	7.43±3.39	5.47±1.96**
BMI (kg/m^2^)	23.40±2.09	29.52±5.50	27.99±10.00**
WHR	0.82±0.049	0.805±0.050	0.93±0.059**
T.Cholestrol(mg/dl)	179.0±18.20	184.53±168.58	188.22±57.62
HDL(mg/dl)	10.71±1.70	11.64±1.93	69.48±86.25**
TG(mg/dl)	85.07±67.28	36.10±31.86	154.32±54.77**
LDL(mg/dl)	167.52±43.7	181.12±169.94	195.30±74.43**
Fibrinogen (ng/ml)	16.22±28.43	4.79±9.52	11.93±37.41**
Exp of Fibrinogen (Arbitory units)	12.03 fold	4.75 fold	5.42 fold**

*p*-value < 0.01** considered highly significance difference among the groups.

**Table 4:** Correlation analysis of Fibrinogen with nephropathological factors in the control, diabetic and diabetic nephropathy group.

**R-values of**	**Groups**	**Serum Creatinine (mg/dl)**	**Blood Urea (mg/dl)**	**Uric Acid (mg/dl)**	**GFR (mL/min/ 1.73m2)**	**BUN (mg/dl)**	**Blood Sugar**
Serum Fibrinogen (ng/ml)	Control	-0.008	-0.016	-0.084	0.014	-0.016	0.056
	DM group	-0.011	0.135**	-0.054	0.042	0.135**	-0.078
	DN group	-0.027	0.003	0.047	-0.013	0.003	0.091
Expression Fibrinogen (fold change)	Control	-0.012	-0.037	-0.072	-0.016	-0.037	-0.06
	DM group	-0.064	-0.057	0.045	0.039	-0.057	-0.002
	DN group	0.026	0.166**	0.033	-0.068	0.166**	0.078

*: Significant Correlation at 0.05 level (two-tailed)

**: Significant Correlation at 0.01 level (two-tailed).

**Table 5a:** Stepwise linear regression, considering GFR as a dependent variable in Control group.

**Variable**	***B***	***95% CI*** ***LL UL***		***SE B***	**β**	***p*-value**	***R^2^***	**Δ*R^2^***	**Sig. F Change**
**Group 1 (GFR)**									
**Model 1**							0.647	0.647	0.000
(Constant)	197.4	191.8	203.01	2.81		0.000	-	-	-
Creatinine (mg/dl)	-137.3	-147.5	-127.1	5.11	-0.80	0.000	-	-	-
**Model 2**							0.685	0.038	0.000
(Constant)	220.10	211.68	228.51	4.278		0.000	-	-	-
Creatinine (mg/dl)	-138.97	-148.64	-129.29	4.919	-0.814	0.000	-	-	-
Age (years)	-0.447	-0.576	-0.318	0.066	-0.196	0.000	-	-	-
**Model 3**							0.693	0.007	0.003
(Constant)	228.35	218.43	238.27	5.044		0.000	-	-	-
Creatinine (mg/dl)	-140.19	-149.80	-130.59	4.885	-0.821	0.000	-	-	-
Age (Years)	-0.444	-0.572	-0.317	0.065	-0.195	0.000	-	-	-
Uric acid (mg/dl)	-1.271	-2.111	-0.443	0.424	-0.086	0.003	-	-	-

**Table 5b:** Stepwise linear regression, considering GFR as a dependent variable in DM group.

**Variable**	***B***	***95% CI*** ***LL UL***		***SE B***	**β**	***p*-value**	***R^2^***	**Δ*R^2^***	**Sig. F Change**
**Group 2 (GFR)**									
**Model 1**							0.818	0.818	0.000
(Constant)	156.94	153.16	160.72	1.925		0.000	-	-	-
Creatinine (mg/dl)	-76.35	-79.97	-72.72	1.845	-.904	0.000	-	-	-
**Model 2**							0.844	0.026	0.000
(Constant)	180.182	173.460	186.891	3.411		0.000	-	-	-
Creatinine (mg/dl)	-76.650	-80.020	-73.291	1.711	-0.908	0.000	-	-	-
Age (Years)	-0.466	-0.581	-0.351	0.058	-0.162	0.000	-	-	-
**Model 3**							0.847	0.003	0.008
(Constant)	169.84	159.69	180.00	5.166		0.000	-	-	-
Creatinine (mg/dl)	-75.71	-79.12	-72.30	1.734	-.897	0.000	-	-	-
Age (years)	-0.465	-0.579	-0.351	0.058	-0.161	0.000	-	-	-
BMI (kg/m^2^)	0.317	0.082	0.552	0.120	0.054	0.008	-	-	-
**Model 4**							0.849	0.003	0.011
(Constant)	169.790	159.701	179.871	5.1282		0.000	-	-	-
Creatinine (mg/dl)	-75.670	-79.060	-72.291	1.722	-0.896	0.000	-	-	-
Age (years)	-0.482	-0.595	-0.368	0.058	-0.167	0.000	-	-	-
BMI (kg/m^2^)	0.318	0.084	0.551	0.119	0.055	0.008	-	-	-
Serum Fibrinogen (ng/ml)	0.173	0.040	0.306	0.068	0.051	0.011	-	-	-
**Model 5**							0.852	0.003	0.008
(Constant)	188.500	171.441	205.561	8.671		0.000	-	-	-
Creatinine (mg/dl)	-76.240	-79.621	-72.850	1.721	-0.903	0.000	-	-	-
Age (years)	-0.443	-0.559	-0.326	0.059	-0.154	0.000	-	-	-
BMI (kg/m^2^)	0.290	0.058	0.523	0.118	0.050	0.015	-	-	-
Serum Fibrinogen (ng/ml)	0.187	0.055	0.319	0.067	0.056	0.006	-	-	-
DBP (mmHg)	-0.232	-0.404	-0.061	0.087	-0.055	0.008	-	-	-
**Model 6**							0.856	0.004	0.001
(Constant)	196.63	179.07	214.18	8.92		0.000	-	-	-
Creatinine (mg/dl)	-74.771	-78.230	-71.310	1.761	-0.88	0.000	-	-	-
Age (years)	-0.467	-.583	-0.351	0.059	-0.162	0.000	-	-	-
BMI (kg/m^2^)	0.277	.047	0.507	0.117	0.048	0.018	-	-	-
Seum Fibrinogen (ng/ml)	0.158	0.027	0.290	0.067	0.047	0.019	-	-	-
DBP (mmHg)	-0.672	-0.989	-0.356	0.161	-0.160	0.000	-	-	-
SBP (mmHg)	0.220	0.086	0.353	0.068	0.128	0.001	-	-	-
**Model 7**							0.858	0.002	0.019
(Constant)	202.85	184.64	221.061	9.262		0.000	-	-	-
Creatinine (mg/dl)	-75.281	-78.741	-71.811	1.760	-0.89	0.000	-	-	-
Age (years)	-0.444	-0.561	-0.327	0.059	-0.154	0.000	-	-	-
BMI (kg/m^2^)	0.262	0.034	0.491	0.116	0.045	0.025	-	-	-
Serum Fibrinogen (ng/ml)	0.150	0.019	0.281	0.067	0.045	0.025	-	-	-
DBP (mmHg)	-0.706	-1.022	-0.390	0.161	-0.168	0.000	-	-	-
SBP (mmHg)	0.218	0.085	0.350	0.067	0.127	0.001	-	-	-
Uric Acid (mg/dl)	-0.447	-0.820	-0.074	0.190	-0.047	0.019	-	-	-
**Model 8**							0.86	0.002	0.041
(Constant)	204.71	186.49	222.93	9.265		0.000	-	-	-
Creatinine (mg/dl)	-75.53	-78.99	-72.07	1.759	-0.894	0.000	-	-	-
Age (years)	-0.464	-0.581	-0.346	0.060	-0.161	0.000	-	-	-
BMI (kg/m^2^)	0.258	0.030	0.485	0.116	0.044	0.027	-	-	-
Serum Fibrinogen (ng/dl)	0.144	0.013	0.275	0.066	0.043	0.031	-	-	-
DBP (mmHg)	-0.700	-1.015	-0.386	0.160	-0.167	0.000	-	-	-
SBP (mmHg)	0.215	0.083	0.347	0.067	0.125	0.002	-	-	-
Uric Acid (mg/dl)	-0.426	-0.797	-0.054	0.189	-0.045	0.025	-	-	-
Expression Fibrinogen	-0.145	-0.283	-0.006	0.071	-0.040	0.041	-	-	-

• Dependent Variable was GFR (mL/min/1.73 m^2^).

**Table 5c:** Stepwise linear regression, considering GFR as a dependent variable in DN group.

**Variable**	***B***	***95% CI*** ***LL UL***		***SE B***	**β**	***p*-value**	***R^2^***	**Δ*R^2^***	**Sig.F Change**
**Group 3 (GFR)**									
**Model 1**							0.673	0.673	0.000
(Constant)	72.074	69.316	74.831	1.400		0.000	-	-	-
Creatinine (mg/dl)	-17.811	-19.111	-16.517	.6634	-0.820	0.000	-	-	-
**Model 2**							0.728	0.055	0.000
(Constant)	87.284	82.921	91.646	2.211		0.000	-	-	-
Creatinine (mg/dl)	-17.671	-18.862	-16.482	0.605	-0.814	0.000	-	-	-
Age	-0.294	-0.362	-0.225	0.035	-0.235	0.000	-	-	-
**Model 3**							0.739	0.004	0.019
(Constant)	89.67	84.98	94.36	2.386		0.000	-	-	-
Creatinine (mg/dl)	-17.84	-19.03	-16.65	.604	-0.822	0.000	-	-	-
Age	-0.287	-0.355	-0.218	0.034	-0.227	0.000	-	-	-
Blood sugar	-0.013	-0.023	-0.003	0.005	-0.065	0.019	-	-	-

• Dependent Variable was GFR (mL/min/1.73 m^2^).

**Table 6:** Demographic and biochemical characteristics of control, diabetes mellitus (DM) and diabetic nephropathy (DN) group in selected cities.

**Parameters**	**Studied groups**	**Lahore** **N=64**	**Gujranwala** **N=64**	**Karachi** **N=64**	**Multan** **N=64**	**Islamabad** **N=64**	**Gharro** **N=64**
**Age (years)**	Control DM DN	49.61±11.54 48.80±11.61 50.80±11.52*	50.72±11.86 52.16±10.48*^b^ 50.95±11.42	44.05±13.77 49.05±11.92 54.62±10.29*^c^	52.70±12.56*^a^ 49.08±10.07 52.26±9.34	47.03±11.65 49.14±10.32 51.65±8.76*	49.25±13.10 49.08±11.25 52.42±11.01*
**HbA1c**	Control DM DN	4.75±0.72 **^a^** 9.30±1.63* 7.85±0.75	4.72±0.77 7.90±0.67 9.40±1.63*^c^	4.68±0.76 9.59±1.61* 7.91±0.69	4.72±0.75 9.61±1.54* **^b^** 7.86±0.68	4.67±0.81 9.61±1.53* 7.98±0.68	4.73±0.75 9.51±1.69* 7.95±0.76
**S.B.P (mmHg)**	Control DM DN	111.09±8.37 131.41±17.53 **^b^** 135.95±15.87*	109.38±8.88 124.44±15.21 138.46±23.73*^c^	114.69±6.89 **^a^** 129.37±18.99* 119.25±18.03	110.31±8.53 127.78±13.25* 90.94±22.31	110.47±8.62 129.68±17.13* 102.66±27.30	109.22±8.78 129.68±16.94* 110.15±31.01
**D.B.P (mmHg)**	Control DM DN	85.00±5.90 83.11±6.72 86.67±7.30*	84.53±6.88 85.38±16.5*^b^ 85.05±9.79	85.94±4.95 83.10±8.15 89.97±18.84*	85.00±6.17 82.03±5.00 120.43±19.06*^c^	85.16±6.42 **^a^** 83.06±7.06 109.22±22.67*	84.22±6.85 82.83±6.73 106.72±22.04*
**Blood Sugar**	Control DM DN	111.31±21.79 262.13±91.79* 131.52±39.9	109.3±21.7 235.22±70.53 252.89±96.32*^c^	112.14±19.77 **^a^** 275.23±80.92* 183.86±66.17	111.14±21.53 288.46±78.28* **^b^** 211.82±73.43	109.84±20.81 276.68±78.08* 188.43±66.17	109.92±2.63 269.25±84.72* 175.35±66.71
**Blood Urea** **(mg/dl)**	Control DM DN	22.25±8.89 43.95±20.30 133.03±35.54*	23.59±9.62 133.25±38.24*^b^ 48.32±13.31	24.13±9.85 44.48±10.93 115.06±26.57*	21.77±8.18 42.08±10.74 125.77±34.64*	25.52±43.03 **^a^** 43.03±12.01 136.08±39.36*^c^	24.09±9.30 43.38±11.97 126.98±34.39*
**Serum Creatinine (mg/dl)**	Control DM DN	0.53 ± 0.15 0.97±0.36 1.93±0.68*	0.53±0.16 1.98±0.55*^b^ 0.90±0.44	0.50±0.18 1.00±0.39 2.02±0.54*	0.48±0.19 1.01±0.36 2.07±0.61*	0.52±0.17 **^a^** 0.98±0.37 2.10±0.62*^c^	0.53±0.14 1.01±0.40 1.95±0.57*
**BUN (mg/dl)**	Control DM DN	10.39±4.15 20.53±9.48 62.16±22.39*	11.02±4.49 62.26±17.87*^b^ 22.58±6.22	11.27±4.60 20.78±5.10 53.76±12.41*	10.17±3.82 19.66±5.01 58.77±16.19*	11.92±4.39 **^a^** 20.10±5.61 63.58±18.39*^c^	11.25±4.35 20.27±5.59 59.33±16.07*
**GFR(mL/min/1.73m^2^)**	Control DM DN	122.88±23.79* 81.77±31.99 40.29±22.39	123.04±28.14* 36.15±10.88 90.19±34.98 **^c^**	131.69±33.49* 79.91±29.95 34.72±13.67	134.19±37.11*^a^ 79.11±31.91 37.09±14.72	124.48±30.09* 83.22±32.01 **^b^** 35.15±14.99	122.07±18.44* 80.29±32.72 39.98±16.17
**Uric Acid** **(mg/dl)**	Control DM DN	6.07 ± 2.00 7.15±3.25* 5.65±1.98	6.09±1.92 5.50±2.04 7.61±3.48*^c^	6.02±2.05 7.41±3.40* 5.49±2.11	6.06±2.03 7.14±3.26* 5.40±1.94	6.12±1.94 **^a^** 7.54±3.47* 5.51±1.90	6.11±1.92 7.64±3.58*^b^ 5.51±1.97
**BMI (kg/m^2^)**	Control DM DN	23.56±2.02 **^a^** 29.93±5.40* 28.37±10.27 **^c^**	23.41±2.01 28.16±10.10* 28.24±5.69	23.20±2.21 30.04±5.47*^b^ 28.34±10.56	23.32±2.16 29.24±5.53* 27.50±9.73	23.55±2.11 29.89±5.51* 28.04±10.00	23.44±2.08 29.96±5.44* 27.76±10.12
**WHR**	Control DM DN	0.84 ± 0.03 0.80±0.04 0.92±0.50*	0.86±0.007 **^a^** 0.95±0.06*^b^ 0.79±0.05	0.77±0.02 0.81±0.05 0.93±0.06*	0.81±0.05 0.79±0.47 0.94±0.06*^c^	0.84±0.03 0.81±0.50 0.92±0.05*	0.82±0.04 0.80±0.05 0.92±0.05*
**T.Cholesterol** **(mg/dl)**	Control DM DN	178.00±181.62 176.15±151.62 188.63±63.99*^c^	179.45±19.81 188.90±56.71* 179.32±148.68	179.53±18.56 192.63±173.2* 186.71±54.50	179.54±16.21 175.89±176.50 185.15±73.84*	177.85±18.83 199.33±209.8*^b^ 187.31±57.12	179.54±17.85 **^a^** 190.60±151.3* 182.41±63.75
**HDL(mg/dl)**	Control DM DN	10.81 ± 1.67 11.74±1.83 71.18±87.65*	10.53±1.79 69.82±86.10*^b^ 11.65±1.98	10.64±1.72 11.71±1.89 67.34±87.11*	10.96±1.59 **^a^** 11.67±1.84 74.37±91.42*^c^	10.47±1.82 11.29±2.05 72.22±86.11*	10.78±1.68 11.83±1.88 66.79±85.04*
**TG(mg/dl)**	Control DM DN	91.12 ± 73.05 37.99±35.54 157.04±60.50*	78.82±60.05 152.35±53.40*^b^ 36.47±34.84	80.51±61.28 37.22±31.85 159.13±57.84*^c^	91.63±75.26 **^a^** 37.30±33.47 136.66±53.94*	79.35±61.91 37.86±33.78 157.07±59.73*	87.12±70.66 32.85±30.01 149.98±55.82*
**LDL(mg/dl)**	Control DM DN	165.63± 46.63 173.81±152.5 189.60±72.00*	172.00±39.52 **^a^** 198.18±75.84*^b^ 174.65±150.18	171.13±40.59 189.46±174.75* 184.23±71.33	162.94±47.12 172.62±177.81 220.55±73.02*^c^	169.88±41.34 196.24±212.55* 186.78±75.73	164.77±46.17 186.43±152.07 191.49±77.13*
**Fibrinogen** **(ng/ml)**	Control DM DN	10.40 ±6.15* 7.33±23.48 2.92±5.65	18.43±47.98* 10.96±6.54 8.74±17.00	18.57±20.37* **^a^** 6.81±8.75 5.60±13.66	3.47±6.32 13.11±35.58 23.29±57.23*^c^	12.18±15.07 3.65±7.28 12.47±45.08*	17.31±21.00* 14.59±35.45 **^b^** 3.23±5.40
**Exp of Fibrinogen** **(Arbitory Units)**	Control DM DN	2.06 fold* 1.48 fold 0.78 fold	46.27 fold*^a^ 15.35 fold **^b^** 10.21 fold	3.83 fold 4.58 fold 5.30 fold*	6.01 fold* 5.25 fold 3.79 fold	6.51 fold 1.92 fold 16.43 fold*^c^	2.39 fold* 1.82 fold 0.74 fold

*: Indicate significant difference in control, DM and DN groups of each city.

**^a^**:Indicate significant difference among control groups of each city.

**^b^**:Indicate significant difference among DM groups of each city.

**^c:^** Indicate significant difference among DN groups of each city.

**Table 7:** Correlation analysis of fibrinogen with nephropathological factor in the control, diabetic and diabetic nephropathy groups of selected cities of Pakistan .

**Variables**	**Selected Cities**	**R value of Serum Fibrinogen**			**R value of Fibrinogen Expression (Fold Change)**		
		**Control Group**	**DM group**	**DN Group**	**Control Group**	**DM Group**	**DN Group**
BUN (mg/dl)	Lahore Gujranwala Karachi Multan Islamabad Gharro	-0.093 -0.075 -0.107 -0.009 0.078 -0.030	0.229 -0.208 0.043 0.285* 0.192 0.179	0.187 0.319* 0.098 0.018 -0.014 -0.005	-0.109 -0.001 0.000 0.061 -0.220 -0.182	-0.131 0.226 -0.048 -0.218 0.034 -0.61	-0.091 -0.021 -0.055 0.257* -0.074 0.065
GFR (mL/min/1.73m^2^)	Lahore Gujranwala Karachi Multan Islamabad Gharro	-0.091 0.123 0.007 -0.123 0.001 0.021	-0.127 -0.070 -0.082 -0.181 -0.251* 0.076	-0.129 0.177 -0.028 0.017 0.026 0.056	-0.124 -0.033 -0.075 -0.001 0.119 -0.010	0.026 -0.077 -0.034 0.110 0.006 0.049	-0.027 -0.078 -0.033 0.145 -0.048 -0.165
Serum Creatinine (mg/dl)	Lahore Gujranwala Karachi Multan Islamabad Gharro	0.047 -0.139 0.093 0.132 0.011 0.065	0.018 0.126 0.083 0.222 0.300* -0.72	0.061 -0.115 -0.075 -0.085 0.002 -0.118	0.073 -0.006 0.183 0.071 -0.100 0.022	0.029 0.040 -0.007 -0.122 0.006 -0.104	-0.084 0.045 0.089 -0.074 0.069 0.167
Blood urea (mg/dl)	Lahore Gujranwala Karachi Multan Islamabad Gharro	-0.093 -0.075 -0.107 -0.009 0.078 -0.030	0.229 -0.208 0.043 0.285* 0.192 -0.179	0.187 0.319* 0.098 0.018 -0.014 0.001	-0.109 -0.001 0.000 0.061 -0.220 -0.182	-0.131 0.226 -0.048 -0.218 0.034 -0.061	-0.091 -0.021 -0.055 0.257* -0.074 0.070
Uric acid (mg/dl)	Lahore Gujranwala Karachi Multan Islamabad Gharro	0.104 0.064 -0.085 0.022 -0.176 -0.010	0.008 -0.133 -0.048 -0.003 0.016 -0.022	0.057 0.013 0.054 -0.052 0.185 0.232	-0.140 -0.157 0.108 0.134 -0.101 -0.032	-0.289* -0.054 0.139 0.089 0.096 0.019	-0.032 0.015 -0.111 0.126 0.043 0.030
Blood Sugar	Lahore Gujranwala Karachi Multan Islamabad Gharro	0.396** -0.278* 0.081 -0.027 -0.123 0.045	0.117 0.072 -0.174 -0.132 -0.126 -0.118	0.060 -0.075 0.406** -0.053 -0.001 -0.029	-0.087 -0.185 0.177 -0.181 0.136 -0.078	-0.078 0.073 -0.054 0.155 0.126 -0.036	0.054 -0.125 0.173 -0.098 0.028 0.056

*: Correlation is significant at 0.05 level (two-tailed)

**: Correlation is significant at 0.01 level (two-tailed).

## Data Availability

All data generated or analyzed during this study are included in this published article.

## LIST OF ABBREVIATIONS

BUN: Blood Urea Nitrogen
BMI: Body Mass Index
DM: Diabetes Mellitus
DN: Diabetic Nephropathy
DHQ: District Headquarter Hospital
GFR: Glomerular Filtration Rate
HbA1c: Glycated haemoglobin
HDL: High Density Lipoproteins
LDL: Low Density Lipoproteins
SBP: Systolic Blood Pressure
DBP: Diastolic Blood Pressure
TG: Triglycerides
T2DM: Type II Diabetes Mellitus
WHR: Wait to Hip Ratio

## STANDARDS OF REPORTING

STROBE guidelines were followed
